# The Outcome of Local Excision of Rectal Adenomas with High-Grade Dysplasia by Transanal Endoscopic Microsurgery: A Single-Center Experience

**DOI:** 10.3390/jcm13051419

**Published:** 2024-02-29

**Authors:** Muhammad Khalifa, Rachel Gingold-Belfer, Nidal Issa

**Affiliations:** 1 Department of Surgery, Rabin Medical Center-Hasharon Hospital, Faculty of Medicine, Tel Aviv University, Petach Tikva 49100, Israel; nidalissa@clalit.org.il; 2 Department of Gastroenterology, Rabin Medical Center-Hasharon Hospital, Tel Aviv University, Petach Tikva 49100, Israel; rachelgingoldbelfer@gmail.com

**Keywords:** transanal endoscopic microsurgery, high-grade dysplasia, rectal polyp

## Abstract

**Background**: Local excision by transanal endoscopic microsurgery (TEM) is considered an acceptable treatment for rectal adenomas with high-grade dysplasia (HGD). This study aims to assess the likelihood of harboring an invasive carcinoma in preoperatively diagnosed HGD polyps and evaluate the risk factors for tumor recurrence in patients with final HGD pathology. **Methods**: Data from patients who underwent TEM procedures for adenomatous lesions with HGD from 2005 to 2018 at the Rabin Medical Center, Hasharon Hospital, were analyzed. Collected data included patient demographics, preoperative workup, tumor characteristics and postoperative results. Follow-up data including recurrence assessment and further treatments were reviewed. The analysis included two subsets: preoperative pathology of HGD (sub-group 1) and postoperative final pathology of HGD (sub-group 2) patients. **Results**: Forty-five patients were included in the study. Thirty-six patients had a preoperative diagnosis of HGD, with thirteen (36%) showing postoperative invasive carcinoma. Thirty-two patients had a final pathology of HGD, and three (9.4%) experienced tumor recurrence. Large tumor size (>5 cm) was significantly associated with recurrence (*p* = 0.03). **Conclusions**: HGD rectal polyps are associated with a significant risk of invasive cancer. Tumor size was a significant factor in predicting tumor recurrence in patients with postoperative HGD pathology. The TEM procedure is an effective first-line treatment for such lesions.

## 1. Introduction

Premalignant colorectal polyps pose a significant risk for cancerous transformation, necessitating timely resection upon diagnosis. Resection has been shown to significantly reduce the risk of cancer development [1].

Among various adenomas, polyps with high-grade dysplasia (HGD) carry the highest risk of transformation into cancer [2,3].

HGD adenomas consist of about 6% of colorectal adenomas and are more likely to be found in the left colon and rectum. According to data analysis from 4763 patients in the National Polyp Study from the US, the likelihood that a particular adenoma harbors high-grade dysplasia relates mainly to adenoma size and the extent of villous component [2].

Ongoing challenges in managing these lesions continue to be identifying risk factors for harboring invasive carcinoma and identifying diagnostic techniques that may aid in deciding appropriate surgical therapy [4,5,6,7,8].

Most of these adenomas are fully resected endoscopically. But some adenomas, particularly large or flat, are not amenable to such resections. In cases where endoscopic resection is not feasible, surgical resection becomes necessary. Segmental resection of the colon is still considered optimal for colonic polyps, while the surgical treatment of rectal polyps has been improved in the last two decades.

The approach to the surgical resection of rectal lesions when endoscopic polypectomy is unsuccessful has historically been radical rectal resection with total mesorectal excision. Alternatively, a number of transanal excision (TAE) methods have proven much less morbid and oncologically safer for benign lesions and early cancer [9]. One such method is transanal endoscopic microsurgery (TEM). TEM is an endoscopic local excision technique, which enables a more precise and high-quality excision of selected rectal lesions [10]. Since its introduction a few decades ago, this technique has proved its superiority over the traditional TAE for benign rectal lesions in terms of the morbidity and quality of specimens [9]. Meanwhile, for early rectal cancer compared to radical surgery, TEM has better functional outcomes and appears to have comparable long-term survival rates [11].

Full-thickness resection by means of TEM and transanal minimally invasive surgery (TAMIS) have emerged as an effective modality for the precise diagnosis and definitive treatment of premalignant polyps and selected early rectal cancers. These techniques have shown comparable outcomes in terms of safety and efficacy [10,11,12,13,14].

When the final pathology following full-thickness resection results in submucosal invasion (T1) with unfavorable features or more advanced rectal cancer, radical rectal resection by total mesorectal excision (TME) with or without neoadjuvant therapy becomes necessary for optimal oncological results [15,16,17,18].

Besides rigid proctoscopy and digital rectal examination, the preoperative workup for premalignant adenomas usually includes an endorectal US (ERUS), aiming to evaluate the level of rectal wall invasiveness, but the role of this expensive test is still questionable regarding its accuracy and additive value for evaluating such lesions [19,20,21].

Follow-up protocols following HGD rectal polyp resection vary among different guidelines. The post-polypectomy surveillance guidelines following the removal of dysplastic lesions frequently lack clarity in distinguishing between rectal and colonic polyps, interpreting the significance of dysplasia within resection margins, assessing the depth of resection and considering whether the resection was conducted through endoscopic or surgical means [22].

The aim of our study is to evaluate HGD rectal polyps in the preoperative and postoperative settings. In the preoperative setup, our objectives include assessing the risk of harboring invasive cancer in apparently HGD polyps and identifying potential preoperative parameters associated with invasive final pathology. Additionally, we aim to evaluate the value of preoperative ERUS in the treatment of such polyps.

In the postoperative setup, our goals are to assess the risk of recurrence following HGD polyp resection using TEM and to identify patient and tumor characteristics associated with recurrence. By addressing these objectives, we aim to enhance the understanding and management of premalignant HGD rectal polyps, ultimately improving patient outcomes and guiding appropriate treatment strategies.

## 2. Materials and Methods

Approval for this retrospective study was obtained from the Institutional Review Board of the Rabin Medical Center (code: 0160-18-RMC, approved on 1 January 2018) with a waiver of informed consent. A retrospective analysis of all patients who underwent TEM procedure for adenomatous lesion with HGD from 2005 to 2018 in Rabin Medical Center, Hasharon Hospital, was conducted. The data collected included patients’ demographic characteristics [age, sex, body mass index (BMI)], preoperative workup including endoscopic and imaging studies, tumor characteristics (size, distance from anal verge (AV), location of rectal wall involvement) and preoperative and postoperative pathological reports (including histological type, largest dimension of the tumor, resection margins, and lympho-vascular involvement).

Follow-up outpatient visits, assessment for recurrence and further treatments and surveillance of patients with rectal cancer were also reviewed.

Patients with history of colorectal cancer or previous systemic chemotherapy were excluded.

The TEM procedure was performed according to the standard technique described by Buess [11]. Patients undergoing TEM were evaluated according to a standard protocol that included clinical examination with digital rectal examination, colonoscopy with biopsy, rigid proctoscopy and endorectal ultrasound (EUS). Both the size and the location of the tumors were determined, as well as their distance from the anal verge (to the lower margin of the tumor). The exact location of the tumor was essential—and was thus assessed—to guarantee that the tumor was facing downward during the surgery.

The preoperative preparation for the patients included mechanical bowel preparation (with polyethylene glycol) on the day prior to the surgery and prophylactic antibiotics (Cefamizine 1 g and metronidazole 500 mg) upon the induction of anesthesia.

All procedures were performed by a single surgeon, with original Richard Wolf (Knittlingen, Germany) TEM equipment, under general anesthesia. The patients were placed in a prone jackknife or lithotomy position, depending on tumor location. The tumor was removed by a full-thickness rectal wall excision with a 1 cm margin. The specimens were pinned and marked for orientation by the surgeon. Rectal defects were closed primarily in a transverse fashion with absorbable sutures.

All patients had a urinary catheter in place at the time of surgery, which was removed the day after. Postoperative pain management included oral dipyrone or paracetamol and oral narcotics (tramadol) for all patients upon demand. Patients were allowed to resume enteral nutrition on postoperative day 1 and were discharged from the hospital 2–3 days after the operation.

The TEM specimens were reviewed by two pathologists separately, before delivering the final pathology report.

Patients were evaluated 2 weeks after their surgery and re-examined at 3-month intervals for the first 2 postoperative years and thereafter by a 6-month follow-up for the next 3 years. Clinical examination and rectoscopy were performed during each of the follow-up sessions.

The preoperatively diagnosed patients with HGD were analyzed as sub-group 1 for risk of harboring invasive carcinoma. Another subset of patients with final pathology of HGD (sub-group 2) was analyzed for risk of tumor recurrence.

Statistical analysis was performed using Statistical Package for the Social Sciences (SPSS) version 22.0. Continuous data were expressed as mean and standard deviation when normally distributed or otherwise as the median and interquartile range (IQR). Categorical data were expressed as numbers and proportions and analyzed using Fisher exact or Chi-Square test. A *p*-value < 0.05 was considered significant.

## 3. Results

**Figure 1:**
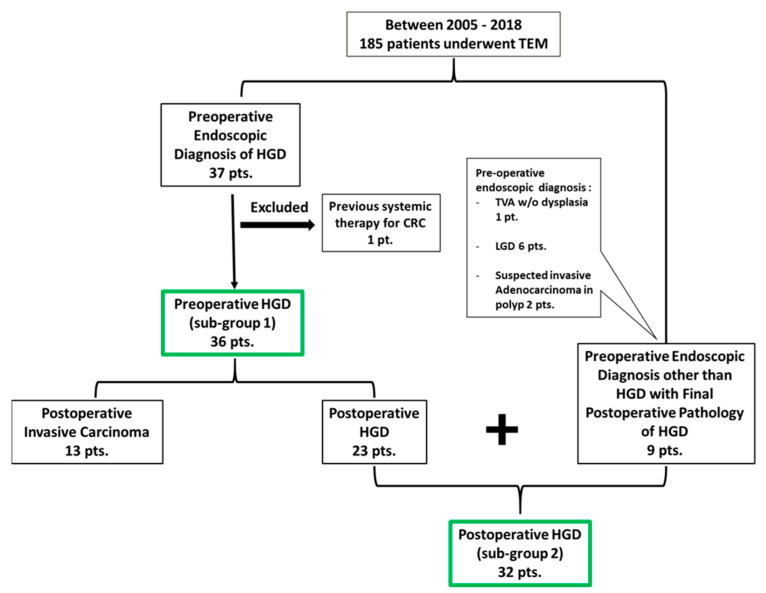
Patient allocation for the study.

Between the years 2005 and 2018, a total of 185 patients underwent a TEM procedure with full-thickness resection for rectal lesions in our institution. Among them, 37 patients were endoscopically pre-diagnosed with sessile adenomatous polyps with HGD. Another nine patients had a final pathology of HGD, while they had a preoperative diagnosis other than HGD. One patient was excluded due to previous systemic chemotherapy for colorectal cancer. A total of 45 patients were included in the study, where the analysis was applied to two subsets: preoperative HGD (sub-group 1) patients *N* = 36 and postoperative final diagnosis of HGD (sub-group 2) patients *N* = 32 (Figure 1).

The mean age of patients was 69 ± 11 years. Twenty-two (49%) patients were male. Moreover, the majority of patients within the study were classified with American Society of Anesthesiologists (ASA) scores of 1–2, tumor size was 3 ± 1.7 cm, and the average distance from the AV was 9 ± 3.5 cm. The mean follow-up period extended over a substantial duration, spanning 60 ± 52 months. Table 1 summarizes patients’ demographics, tumor characteristics and follow-up period.

### 3.1. Preoperative HGD (Sub-Group 1) Analysis

Thirty-six patients had a preoperative endoscopic diagnosis of HGD. Among them, 13 patients (36%) had a final postoperative pathology of invasive carcinoma. All the specimens were R0 resections according to the final pathology report. The tumor grading was well differentiated in 11 (85%) and moderately differentiated in 2 patients. The tumor invasiveness of the rectal wall was T1 in nine patients, T2 in three and T3 in one patient. There was no lympho-vascular invasion in any of the 13 carcinomas, and there were 3 mucinous tumors.

Preoperative tumor characteristics including tumor size, villous histology and distance from the AV were analyzed for the risk of harboring invasive carcinoma, with no statistical significance found.

Table 2 summarizes the tumor parameters according to the final pathology and analysis of the risk of harboring invasive carcinoma.

All the patients with T1 cancer were closely followed up with no further intervention. The remaining three patients with T2 cancers underwent an immediate salvage total mesorectal excision, and the one patient with T3 proceeded to neoadjuvant chemoradiation therapy followed by TME.

Twenty-three out of the thirty-six patients (63.8%) had a preoperative ERUS. The accuracy of the test for diagnosing Tis and T1 cancer was very low, 43% and 30% respectively. Only one patient had an ERUS staging of T2 and happened to have a T1 on the final pathology. The ERUS did not change any treatment plan, and no TEM procedure was upgraded to radical resection depending on the ERUS results.

### 3.2. Postoperative HGD (Sub-Group 2) Analysis

Thirty-two patients had a final pathology of HGD with free-of-tumor resection margins (R0). Three (9.4%) patients had a tumor recurrence in the follow-up period. All three recurrences were pre-diagnosed as HGD and proceeded to the close-follow-up protocol after the TEM procedure. One patient had HGD local recurrence after a 12-month follow-up and proceeded to the re-TEM procedure, with no evidence of recurrence in a 5-year follow-up period. One other patient had invasive carcinoma local recurrence after 24 months and proceeded to low anterior resection with TME. No further recurrence was diagnosed in a 5-year follow-up period. The last patient had a local recurrence of invasive carcinoma after a 60-month follow-up period. He was treated with neoadjuvant chemoradiation therapy followed by TME. He was diagnosed with distant lung metastasis one year later.

The risk of tumor recurrence was assessed according to postoperative tumor characteristics which included tumor size, resection margins and tumor location with respect to the AV. We found a significant relation between large tumors (>5 cm) and the risk of recurrence (*p* = 0.03).

There was no statistical significance regarding the tumor resection margins, the distance from AV and the risk of local recurrence (Table 3).

Table 4 summarizes all the cases of recurrence.

## 4. Discussion

Local excision with full-thickness excision has been accepted widely as a first-line treatment for assumed benign rectal polyps not amenable to endoscopic resection [23,24].

The incidence of malignant invasive carcinoma on the final pathology has been described as 10–20% of the cases [4,6], with a higher risk in cases of large polyps, villous histology and a degree of dysplasia [2,25,26,27].

TEM with full-thickness excision can not only provide a precise histopathological evaluation for treatment guidance, but it can also afford curative treatment for all of the benign approved polyps and selected early T1 cancers that have no high-risk features for nodal involvement, such as poorly differentiation histology, lympho-vascular invasion or deep submucosal invasion [28,29].

Several authors [10,11,12,13,14,15,16,17,18] have shown that TEM, when compared to radical rectal resection by total mesorectal excision, was a safe and effective treatment tool for benign rectal lesions and low-risk T1 rectal cancers, with a lower morbidity rate and acceptable recurrence rate [30,31,32]. Nonetheless, little focus in the literature has been on rectal polyps with HGD. In our study, we evaluated this sub-group of polyps taking into account the reliability of the endoscopic pathology and preoperative workup and the postoperative risk of recurrence.

Among our group of patients who had apparent HGD polyps preoperatively, 35% appeared to have invasive carcinoma on the final pathology with free margins in all of the specimens. We could not find a significant relation between the polyp size or location along the rectum and the risk of harboring invasive cancer. While certain publications have posited an interrelation between adenoma size and the likelihood of invasive carcinoma inclusion [6,27], contrasting findings from other investigations have failed to establish a statistically significant association [23,24]. In the series of Rameriz et al., on the local full-thickness excision of sessile rectal polyps, out of 173 patients with assumed benign polyps who underwent TEM, 14% had an invasive carcinoma in the final pathology, and no statistically significant relation was found between the size or location of the polyp and the risk of harboring cancer [23]. Along with our findings, it seems that the first-line treatment advised for these polyps is full-thickness local excision, for accurate diagnosis and to inform decisions on further management. The high incidence of invasive carcinoma in our study is probably related to the high grade of dysplasia being an independent risk factor for malignant polyps.

We also evaluated the role of preoperative ERUS in the settings of assumed HGD diagnosis and could not find any additive value for such a test in these settings. Of the 23 patients who underwent preoperative ERUS, the test over-staged 16 patients (70%) with assumed preoperative ERUS diagnosis of invasive carcinoma. This can mislead treatment and lead to unnecessary radical surgery. 

Several reports in the literature have raised the question about the accuracy of preoperative ERUS in evaluating villous adenomas and found it to be inaccurate [19,20,21]. Similar to our results, Letarte et al., in their series, found that of the 22 patients who had undergone preoperative ERUS, 11 (50%) were over-staged while 2 (9%) were under-staged [21]. Despite the limiting retrospective nature and small sample size of the study, the need for preoperative ERUS in the settings of clinical and endoscopic preoperative diagnosis of villous adenoma, before proceeding to TEM, is highly questionable. Moreover, the proposition of radical surgery for cases lacking definitive evidence of invasive malignancy carries a substantial risk of overtreatment. In situations where it is feasible to conduct a local excision, thereby facilitating a conclusive diagnosis and, in numerous instances, achieving a curative outcome in a single intervention, such conservative management strategies warrant careful consideration. Based on that, proceeding to full-thickness local excision following clinical and endoscopic diagnosis seems to be the most effective method. However, it is imperative to underscore that further research endeavors, encompassing larger sample sizes, are imperative to furnish a more definitive assessment of this method’s efficacy.

Of the 13 patients with a final pathology of invasive carcinoma after TEM, 9 (70%) were characterized as having T1 tumors without adverse prognostic indicators, thereby rendering the TEM procedure potentially curative in these instances. Regrettably, within this cohort, a single patient experienced recurrence within one year post-TEM, culminating in local and distant metastases and eventual mortality attributable to metastatic disease. Notably, this specific patient exhibited mucinous tumor histology, which prompts the hypothesis that mucinous tumors may introduce an additional risk factor for lymph node involvement and subsequent local recurrence [33]. Consequently, there arises a question as to whether full-thickness local excision suffices as an adequate therapeutic approach in the context of such histopathological entities. Further comprehensive investigations in this domain are warranted.

The other 30% of cancers were T2 and T3 tumors, which proceeded to radical TME, with no recurrence reported during the follow-up period. Although it is a very small number of cases, subsequent TME following local excision has been approved to be safe with acceptable oncological outcomes in several studies [34,35,36], unlike delayed salvage TME following recurrence [37,38].

Within the cohort of patients with a conclusive histopathological diagnosis of high-grade dysplasia (HGD), a recurrence was observed in three (9%) patients, with two (6%) of them experiencing malignant local recurrences. This recurrence rate closely aligns with the reported risk range of 4% to 10% documented in the extant literature [39,40]. Our investigation revealed a statistically significant association (*p* = 0.03) between the presence of very large polyps (>5 cm) and an elevated risk of recurrence over a 60-month follow-up period. These findings corroborate those of a larger-scale study conducted by Scala et al., wherein 320 patients underwent TEM for rectal adenomas, with 113 patients presenting giant adenomas (>5 cm) and demonstrating a significantly higher risk for local recurrence within a 40-month follow-up duration [24].

These observations could potentially be attributed to the presence of residual microscopic disease or free tumor cells disseminated during the procedure, which appears to be more prevalent in cases involving giant adenomas, despite the presence of a pathological report indicating tumor-free resection margins. Given this heightened risk of recurrence in giant polyps, it raises the question of whether such cases may benefit from a more aggressive approach, such as radical surgery. Also, the non-tumor touch technique may minimize cell dissemination.

We did not observe a statistically significant correlation between the distance of the tumor resection margins or the proximity to the AV and their influence on the risk of recurrence. While it is well established that R1 resections represent an independent risk factor for recurrence [23,24], it is noteworthy that all specimens in our series were classified as R0 resections, and we did not identify a significant impact of the closest tumor distance on the risk of recurrence.

In light of these findings and in consideration of previously well-established research concerning the occurrence of local recurrence, we endorse a rigorous surveillance protocol following the resection of premalignant tumors, particularly in cases involving giant polyps. Our surveillance protocol post-TEM surgery, for dysplastic and malignant polyps, encounters patient evaluation 2 weeks after the surgery, followed by re-examination at 3-month intervals for the first 2 postoperative years and every 6 months thereafter for the next 3 years.

It is important to acknowledge several limitations in our study. The retrospective nature of the study introduces potential biases in data collection and selection. Additionally, the relatively small sample size may limit the statistical power and precision of our findings. Moreover, the extended study period spans several years, potentially reflecting changes in clinical practices and diagnostic methods. As such, our results should be interpreted with caution. Further research with larger, more contemporary cohorts is needed to validate and expand upon our findings.

## 5. Conclusions

High-grade dysplastic rectal polyps are associated with a substantial risk of containing invasive carcinoma, necessitating their resection using a full-thickness en bloc resection approach to facilitate subsequent optimal therapeutic interventions. TEM remains a favored primary treatment modality for such lesions, owing to its accessibility, safety profile and effectiveness. It is worth noting that giant high-grade dysplastic rectal polyps carry a noteworthy risk of recurrence, thereby mandating a stringent postoperative surveillance regimen. The consideration of radical surgery as the optimal treatment for these sizable polyps warrants further investigation.

## Figures and Tables

**Table 1:** Patient demographics, preoperative workup, tumor characteristics and follow-up.

*N* = 45	
Age	69 ± 11
Male	22 (49)
ASA score	
1–2	29 (64)
3–4	16 (36)
Preoperative tumor characteristics	
Villous component (*n*, %)	25 (55%)
Largest dimension (cm)	3 ± 1.7 (0.6–8)
Distance from AV (cm)	9 ± 3.5 (2–18)
Location (%)	
Anterior	12 (27)
Posterior	12 (27)
Right	8 (17)
Left	12 (27)
NR	1 (2)
Preoperative imaging (%)	
ERUS	23 (51)
CT	3 (7)
MRI	2 (2)
Follow-up, mean, months	60 ± 52

**Table 2:** Tumor characteristics on final pathology and risk of invasive carcinoma (sub-group 1).

Preoperative HGD (Sub-Group 1) *N* = 36	
Final Pathology	Value
HGD	23 (64%)
Invasive Carcinoma	13 (36%)
Well Diff.	11 (85%)
Mod Diff.	2 (15%)
Poor Diff	0
T1	9/13 (69%)
T2	3/13 (23%)
T3	1/13 (8%)
LVI	0
Mucinous	3 (23%)
Risk of Invasive Carcinoma	*p* Value
Tumor Size > 3 cm	0.73
Distance from AV < 6	1
Villous Component	0.26

**Table 3:** Postoperative analysis for HGD on final pathology and risk of recurrence, sub-group 2 *N* = 32.

HGD on Final Pathology	Recurrence (*n*)	*p* Value
Tumor Size		
>5 cm	2	0.03
<5 cm	1	
Margins		
≤3 mm	0	0.99
>3 mm	3	
Distance from AV		
≤6 cm	2	0.18
>6 cm	1	

**Table 4:** Characteristics of patients with recurrence.

Patients	Endoscopic Pathology	Final Pathology	Tumor Size	Distance from AV	Margins	Recurrence	Intervention	Follow-up
Pt. 1	HGD	HGD	2 × 1.8 cm	5 cm	>5 mm	Local recurrence after 12 months, HGD	Re-TEM	No recurrence in 6 years
Pt. 2	HGD	HGD	7 × 5.5 cm	10 cm	>5 mm	Local recurrence after 24 months, well-mod. AC	LAR with TME	No recurrence in 5 years
Pt. 3	HGD	HGD	6 × 5.8 cm	6 cm	>5 mm	Local recurrence after 60 months, invasive carcinoma	Neoadjuvant CRT followed by TME	Recently lung mets and suspected local recurrence. On biologic treatment

## Data Availability

The data that support the findings of this study are available from the corresponding author, M.K., upon reasonable request.
